# Astragalus membranaceus up-regulate Cosmc expression and reverse IgA dys-glycosylation in IgA nephropathy

**DOI:** 10.1186/1472-6882-14-195

**Published:** 2014-06-18

**Authors:** Ling Ji, XiaoLei Chen, Xiang Zhong, Zi Li, Lichuan Yang, Junming Fan, Wanxing Tang, Wei Qin

**Affiliations:** 1 Division of Nephrology, Department of Medicine, West China Hospital of Sichuan University, 37 # Guoxue road, Wuhou District, Chengdu, Sichuan, China; 2 Division of Nephrology, Sichuan Provincial People’s Hospital, Chengdu, Sichuan, China; 3 State Key Laboratory of Biotherapy of Sichuan University, Chengdu, Sichuan, China

**Keywords:** IgA nephropathy, Astragalus membranaceus, Cosmc, Glycosylation

## Abstract

**Background:**

Decreased Core I β3-Gal-T-specific molecular chaperone (Cosmc) expression induced IgA1 aberrant glycosylation is the main characteristic of IgA nephropathy (IgAN). This study tried to elucidate the effect of Astragalus membranaceus on Cosmc expression and IgA O-glycosylation of peripheral B lymphocytes in IgAN patients.

**Methods:**

Peripheral B lymphocytes of 21 IgAN patients and 10 normal controls were isolated and cultured with or without lipopolysaccharide (LPS) and Astragalus membranaceus injection (AMI). Cosmc mRNA and protein expression levels were measured by real-time RT-PCR and Western blot. IgA1 and glycosylation level were determined by enzyme-linked immunosorbent assay (ELISA) and VV lectin-binding method.

**Results:**

Cosmc mRNA expression and IgA1 O-glycosylation level in IgAN patients was significantly lower than normal controls at baseline. Treatment of LPS could obviously inhibit Cosmc expression and increase the IgA1 secretion in peripheral B lymphocytes of IgAN patients, which resulted in a significantly increase in IgA1 aberrant glycosylation level. Addition of AMI could remarkably up regulated Cosmc expression, decrease IgA1 secretion, and reverse glycosylation level in a dose related manner.

**Conclusion:**

AMI can up-regulate Cosmc expression of peripheral B lymphocytes and reverse IgA1 aberrant O-glycosylation level, which might be the underlying mechanism of AMI therapy in treating IgAN.

**Trial registration:**

TCTR20140515001 (Registration Date: 2014-05-15)

## Background

IgA nephropathy (IgAN) is one of the most common glomerulonephritis in the world, accounts for >50% of biopsy-proven primary glomerulonephritis in Asia, especially in China [1,2]. Recent investigations indicated that abnormalities of IgA1 O-glycosylation induced by decreased Cosmc (core I β3-Gal-T-specific molecular chaperone) expression may be one of the key pathogeneses of IgAN [3]. Reversing of Cosmc expression and aberrant IgA1 O-glycosylation may be a potential treatment of IgAN.

Astragalus membranaceus (AM) is a traditional Chinese herb, which is widely used in treating various renal diseases, including IgAN [4,5]. Many studies demonstrated to that AM have therapeutic effects on reducing proteinuria, reversing hyperlipidaemia, regulating auto-immunity and protecting kidney function in vitro and in vivo. However, the underlying molecular mechanism of its effect in treating IgAN is far from clear. In the present study we aimed to elucidate whether the up-regulation of Cosmc expression and reversing of IgA1 dys-glycosylation are underlying mechanisms of therapeutic action of Astragalus membranaceus in IgAN.

## Methods

### Patients and normal controls

Twenty-one biopsy-proven IgAN patients were included in this study. Diagnosis of IgAN was based on the manifestation of generalized glomerular mesangial proliferation with the presence of IgA as the sole or predominant immunoglobulin deposition in mesangial area of glomeruli. Patients had never received corticosteroids or other immunosuppressive therapy. Patients with systemic diseases such as Schonlein–Henoch purpura, rheumatoid arthritis, diabetes mellitus or liver cirrhosis were excluded. Ten age and sex matched healthy volunteers were selected as normal controls. Measurements of blood pressure (BP), urine routine and serum creatinine were performed to exclude those who had abnormal findings. All of the patients and healthy controls were from Chinese Han nationality.

This study was approved by the ethics committee of West China Hospital of Sichuan University according to the Declaration of Helsinki. Written informed consent approved by the ethics committee was collected from every subject involved in this study. This study is registered as TCTR20140515001 in (Registration Date: 2014-05-15) Thai clinical trial center.

### Drugs

Astragalus membranaceus injection (AMI) was produced by Chengdu Diao Pharmaceutical Company (1 ml AMI is equivalent to 2 g crude drug) and diluted with RPMI 1640 medium to 2 g/ml. LPS was purchased from Sigma Company, which was dissolved in RPMI 1640 medium to 500 μg/ml. AMI solution and LPS solution stored at 4°C for later cell culture.

### Lymphocyte isolation

Lymphocytes were obtained following a previously reported method [6]. Briefly, 20 ml venous blood sample was collected in EDTA anticoagulated tubes. Peripheral blood mononuclear cells (PBMCs) were separated by density gradient centrifugation using Lymphocyte-H lymphocyte isolation media (Cedarlane Laboratories Limited, Canada). PBMCs were washed 3 times with Phosphate Buffered Saline (PBS, Sigma, USA) and resuspended in PBS + 2% Fetal bovine serum (FCS, GIBCO, USA). Peripheral B lymphocytes were then isolated using EasySep Human CD19 Selection Kit magnetic beads (Stem cell, USA) according to manufacturer’s protocol. The purity of B lymphocyte was greater than 95% by by flow cytometry analysis (BD Bioscience, USA). Freshly isolated B lymphocyte of each sample was resuspended in RPMI-1640 medium (10^7^cells/ml, Gibco, USA). Cell morphology was monitored using a phase contrast microscope and cell viability was detected by trypan blue dye staining which showed that cell activation > 95%.

### Lymphocyte culture and treatment

Isolated B Lymphocytes were cultured (10^4^ cells/ml) with complete RPMI-1640 medium containing 15% fetal calf serum + L-glutamine 2 mM, HEPES 1 mM, penicillin 100 U/mL, streptomycin 100 mg/mL in 24-well plates at 37°C. Lymphocytes were divided into four groups: Group A (Baseline): Baseline; Group B (LPS): RPMI + LPS; and Group C (Low AMI): RPMI + LPS + Low dose AMI; Group D: (High AMI): RPMI + LPS + High dose AMI. The concentration of LPS was 12.5 μg/mL. Low and High dose AMI were 200 mg/mL and 1000 mg/mL, respectively. Cells were cultured in 24-well plates at 37°C for 3 days. Viability of B lymphocytes was about 85% as determined by trypan blue.

### IgA1 ELISA analysis and Vicia villosa lectin-binding assay

IgA1 concentration of cell culture supernatants were determined by ELISA [7]. As previously described, 96-well plates were coated with primary antibody (Southern Biotechnology Associates, USA) overnight at 4°C. After blocking, samples were added in duplicate and incubated for 1 h at 37°C with biotinylated secondary antibody (Southern Biotechnology Associates, USA) and peroxidase-avidin D (Vector Laboratories, UK), separately. Thereafter, color was developed using tetramethyl benzidine dilution (TMB) and detected at 450 nm. Standard curve constructed with a serial dilution of IgA1 standard serum (Nordic Immunological Laboratories, Netherlands) was used to calculate IgA1 concentration. The levels of IgA1 O-glycosylation were determined by Vicia villosa (VV) lectin-binding assay. Briefly, samples were added in duplicate to 96-well plates coated with primary antibody and incubated with biotinylated VV lectin (Vector Laboratories, UK) at 37°C and peroxidase-avidin D (Vector Laboratories), color was developed and detected as above.

### Cosmc gene qPCR quantification

Total RNA was extracted from lymphocytes using the RNeasy Mini kit (QIAGEN, USA). Real-time quantitative PCR (RT-PCR) was performed with the Taqman probe technique after reverse transcription. The primers and probes of Cosmc and GAPDH (internal control) are listed in Table 1 (synthesized by Invitrogen, China). The PCR reaction was performed in a Roche Lightcycler (Roche Diagnostics, USA) as previously described. In order to examine the efficiency of RT-PCR, standard curves were established with serial dilutions of sample RNA (500 ng; 10 × dilution). PCR products were purified and sequenced directly (Invitrogen, China). The sequencing result showed that the amplified fragment was in accordance with the GenBank record.

**Table 1:** Primers and fluorescence probes

	**Cosmc**	**GAPDH**
**Sense**	5-GTAACGGAGTGGTGCGCCAA-3	5-GGGTGTGAACCATGAGAAGT-3
**Antisense**	5-TTGCACTTCATCCGCGTCTAGA-3	5-CCAAAGTTGTCATGGATGACCT-3
**Probe**	5-FAM-CGTGCGCGGCTGCGCTTTCCT-TAMRA-3	5-FAM-CTGCACCACCAACTGCTTAGC-TAMRA-3

### Cosmc protein quantification

Cosmc protein quantification was performed using Western blot as previously reported [8]. Protein samples of lymphocytes were separated on 10% SDS PAGE gels and transferred to PVDF membranes. After blocking, blots were incubated with primary antibodies (1:200; Santa Cruz Biotechnology, USA) overnight at 4°C and then with horseradish peroxidase-linked secondary antibodies (1:5000; Santa Cruz Biotechnology, USA). After further washing, the immunoreactivities of antibodies were detected via ECL reagents (GE Healthcare, USA). The measurement of GAPDH was applied as an internal calibrator. OD ratio of Cosmc/GAPDH was measured and analyzed using Image-J software.

### Statistical analysis

Student t-test analyses were performed to evaluate the changes in IgA1 and VV lectin binding levels. Delta Ct was used in the analysis of Cosmc mRNA qPCR analysis. P-value of 0.05 was taken as the level of statistical significance. Pfaffl’s method, the most accepted method of qPCR analysis, was applied during the analysis of real-time PCR results.

## Results

### General features of subjects included

The clinical characteristics of the subjects are shown in Table 2. Measurements of BP, urine routine, serum creatinine, 24 hour urinary protein quantification were performed in all patients. No significant difference was observed in the age, sex and ethnological background of IgAN patients and normal controls included (P > 0.05).

**Table 2:** Baseline clinical characters of IgAN patients

	**IgAN (n = 21)**	**Control (n = 10)**
Age (years)	27.53 ± 9.38*	29.5 ± 4.2*
Males/females	11/10	6/4
Disease duration (months)	14.12 ± 23.28*	N/A
Blood Pressure systolic (mmHg)	117.6 ± 15.8*	108.5 ± 15.5*
Blood Pressure diastolic (mmHg)	75.5 ± 11.6*	65.5 ± 6.8*
Proteinuria (g/24 hr)	2.8 ± 1.7	N/A
Serum Creatinine (μmol/L)	89.3 ± 35.6*	N/A

*Expressed as mean ± standard deviation.

### Effects of AMI on peripheral B lymphocyte IgA1 secretion

**Figure 1:**
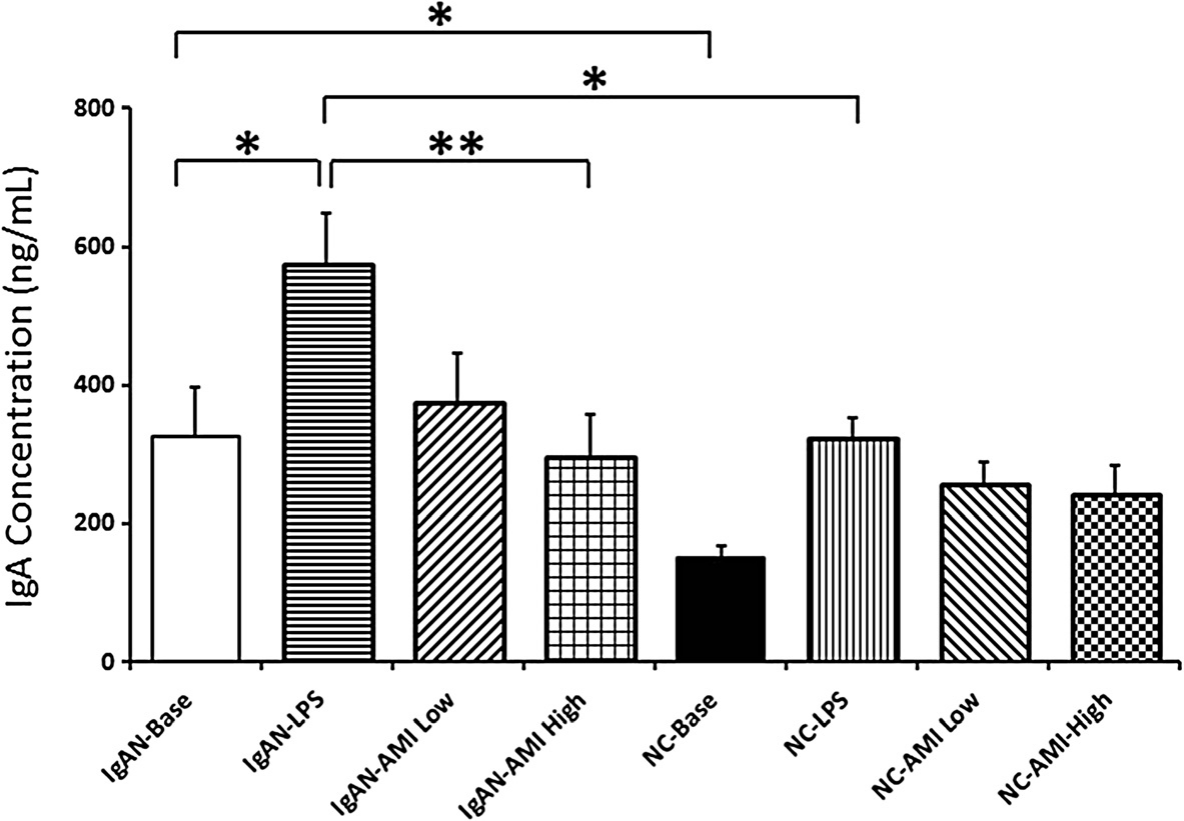
**IgA1 (ng/mL) concentration in supernatant from IgAN patients and normal controls peripheral B lymphocytes.** After LPS stimulation, IgA1 was remarkably increased, especially in IgAN group. Treatment with AMI could inhibit the secretion of IgA1.

In cultured B lymphocyte from IgAN patients, IgA1 concentration in culture medium increased to a dramatically higher level than in normal controls after treatment of LPS. However, Astragalus membranaceus injection (AMI) treatment could apparently inhibit the IgA1 secretion. A dose related inhibitory effect of AMI on IgA1 secretion was observed. No significant effect of AMI on IgA1 secretion in normal controls was observed (Figure 1, Table 3).

**Table 3:** IgA1 concentration (ng/mL) in IgAN patients and normal controls

	**IgAN (n = 21)**	**Normal controls (n = 10)**
Baseline	326.04 ± 71.44^a,b^	150.73 ± 17.78^a^
LPS	573.86 ± 73.84^b,c,d^	322.89 ± 30.32^d^
LPS + AMI (Low)	376.12 ± 69.94	255.93 ± 33.64
LPS + AMI (High)	295.51 ± 61.75^c^	241.14 ± 42.68

^a,b,c,d^comparison between 2 groups p < 0.05.

### Effects of AMI on peripheral B lymphocyte IgA1 glycosylation

**Figure 2:**
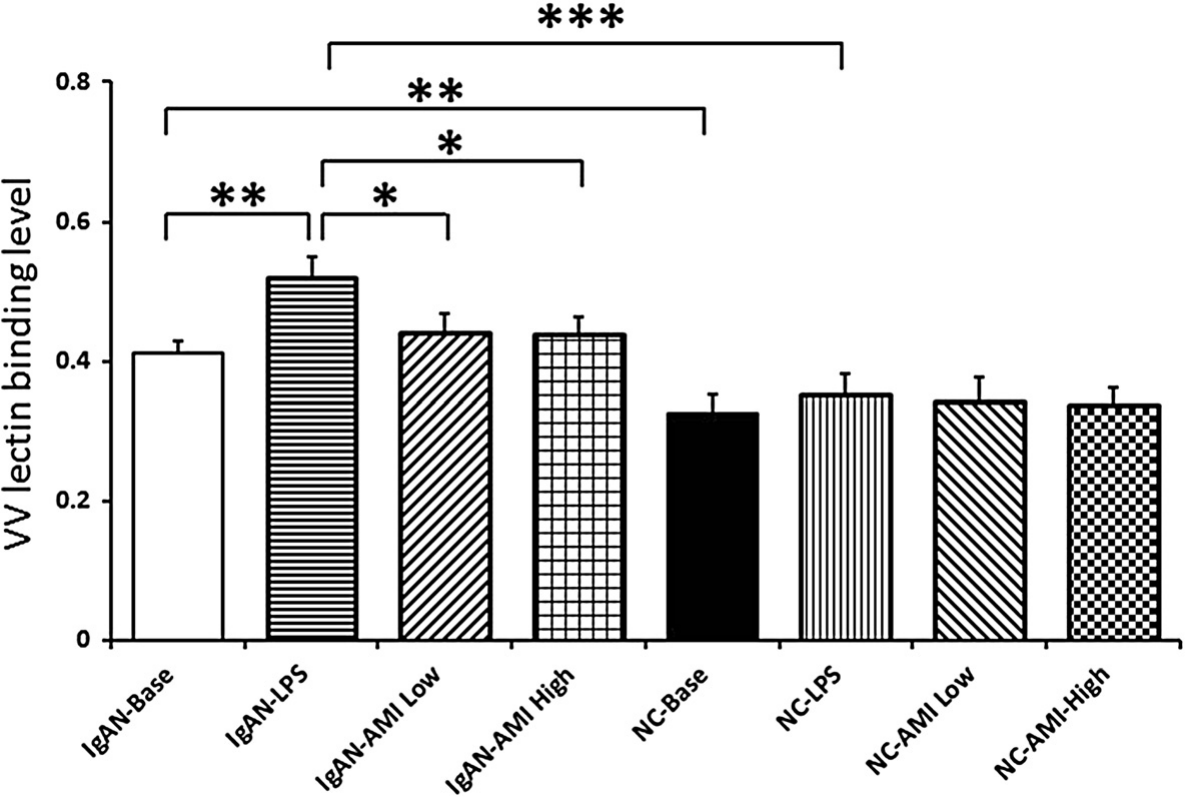
IgA1 O-glycosylation assay in supernatant of IgAN patients and normal controls peripheral B lymphocytes.

Baseline serum VV lectin binding level of IgAN patient was significantly higher than that of normal control, indicating a higher aberrant glycosylation level. After LPS stimulation, the VV lectin binding level dramatically increased. Adding of AMI could reverse the increase of VV lectin binding level, which indication an improvement of dys-glycosylation level of IgA1 secreted by cultured B lymphocytes of IgAN patients (Figure 2, Table 4).

**Table 4:** IgA1 VV lectin binding level in IgAN patients and normal controls

	**IgAN (n = 21)**	**Normal controls (n = 10)**
Baseline	0.41 ± 0.02^a,d^	0.32 ± 0.03^d^
LPS	0.52 ± 0.03^a,b,c,e^	0.35 ± 0.03^e^
LPS + AMI (Low)	0.44 ± 0.03^b^	0.34 ± 0.03
LPS + AMI (High)	0.44 ± 0.02^c^	0.34 ± 0.02

^a,b,c,d,e^comparison between 2 groups p < 0.05.

### Effects of AMI on Cosmc gene expression

**Figure 3:**
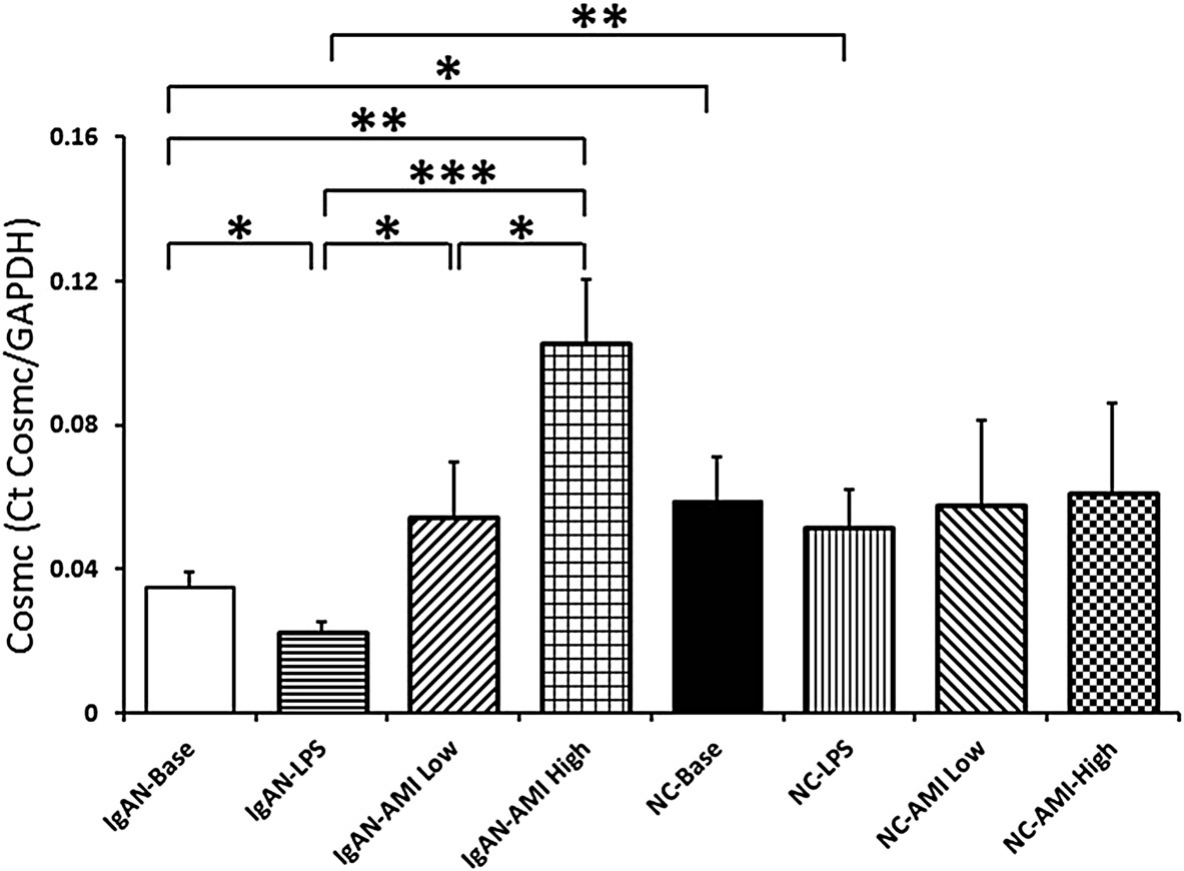
The expression levels of Cosmc mRNA represented by Delta Ct.

**Figure 4:**
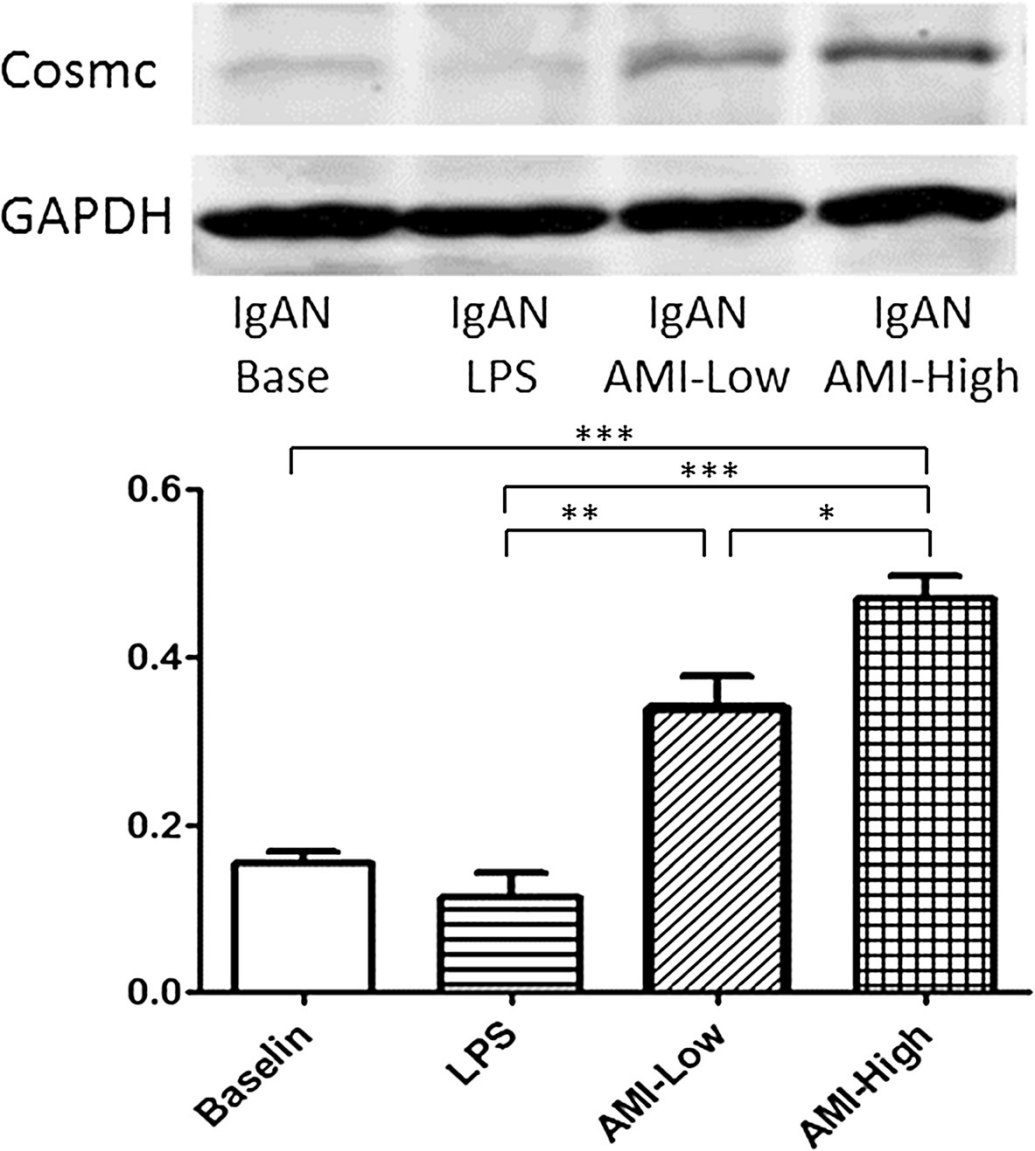
Western blot analysis for Cosmc protein after cultured in presence or absence of LPS and AMI.

The baseline Cosmc mRNA expression level in peripheral B lymphocytes of IgAN patients was significantly lower than that of normal controls. Co-culture with LPS decreased the Cosmc mRNA expression dramatically. Addition of AMI could increase the mRNA expression of Cosmc gene apparently in a dose related manner. (Figure 3, Table 5). Western blot was applied to measure the Cosmc protein expression levels in each group, which indicated a result in accordance with that of qPCR results (Figure 4).

**Table 5:** Cosmc mRNA level in IgAN patients and normal controls

	**IgAN (n = 21)**	**Normal controls (n = 10)**
Baseline	0.035 ± 0.005^a,e,f^	0.059 ± 0.013^f^
LPS	0.023 ± 0.003^a,b,c,d,g^	0.052 ± 0.011^g^
LPS + AMI (Low)	0.055 ± 0.015^b^	0.058 ± 0.024
LPS + AMI (High)	0.103 ± 0.018^c,d,e^	0.061 ± 0.025

^a,b,c,d,e,f,g^comparison between 2 groups p < 0.05.

## Discussion

IgAN is the most common glomerulonephritis in Asian countries. Hinge region dys-glycosylation of IgA1 molecule is considered as one of the key pathogenesis of IgAN. It was reported that sera, mesangial deposited and tonsil secreted IgA1 molecules in IgAN were significantly low in O-glycan level [9,10]. In previous studies we found that the specific functional chaperon of β1,3-galactosyltransferases, which participates in the glycosylation process of IgA1, is remarkably lower in peripheral B lymphocyte of IgAN patients [3,6]. Moreover, reverse of Cosmc gene expression might be the potential mechanism of mycophenolic acid in treating IgAN [7,8].

Astragalus mongholicus (AM) derived from the dry root of Astragalus membranaceus, which belongs to leguminous plant of the Astragalus family, is one of the most popular traditional Chinese medicines. AM is composed of glycoside, astragalus polysaccharides, multi-amino acids, astragalus total saponin, caritinoid, astragalus total flavonoids, and microelement. It has been widely used in China and East Asia area for many years to treat myocardial ischemia, liver fibrosis, chronic nephritis, diabetes, etc. [4,11]. It has been shown to have several biological properties: antioxidant, antiaging, antiviral and inhibiting intracellular calcium overload. AM has been shown to possess renoprotective activity by attenuating glomerular sclerotic injury in experimental diabetic nephropathy. Previously, we found that AM could significantly inhibit the renal fibrosis by up-regulating hepatocyte growth factor (HGF) and down-regulating transforming growth factor-β1 (TGF-β1) [12]. Further study indicated that AM could lower the level of hematuria, 24 hours-albuminuria and urine NAG of the IgAN model, and amelioratse the change of the renal pathology and reduce the deposit of IgA in glomerular mesangium [5]. Down-regulation of NF-kappaB and MCP-1 expression in kidney as well as regulate the balance of Th1 and Th2 cells was considered as the potential mechanism of AMI in treating IgAN [13,14]. However, no study has been done to clarify the effect of AMI on dys-glycosylation of IgA1 in IgAN patients.

In the current study, we found that peripheral B lymphocytes from IgAN patients secreted apparently higher level of IgA1 compared with normal controls at baseline and under LPS stimulation. VV lectin binding assay indicated significant aberrant IgA1 O-glycosylation in IgAN patients. qPCR shown deficiency of Cosmc gene expression in IgAN patients. These data were in accordance with our previous findings [3,6].

Previously, we found that reverse of Cosmc expression was a potential method of treating IgAN. It was found that up-regulation of Cosmc expression by 5-AZA and MPA could reverse the IgA1 dys-gylcosylation level significantly [7,8]. In this study, we found that treatment with AMI could dramatically inhibit the secretion of IgA1 induced by LPS stimulation in peripheral B lymphocytes from IgAN patients (573.86 ± 73.84 vs 376.12 ± 69.94 vs 295.51 ± 61.75, p < 0.05). Meanwhile, the expression levels of Cosmc increased signicantly after addition of AMI in a dose dependent manner (0.023 ± 0.003 vs 0.055 ± 0.015 vs 0.103 ± 0.018, p < 0.05). These changes indicated an apparently improvement of O-glycosylation in IgA1 secreted from isolated peripheral B lymphocytes (0.52 ± 0.03 vs 0.44 ± 0.03 vs 0.44 ± 0.02, p < 0.05). These results suggested that, reverse of Cosmc gene expression and improve of IgA1 O-glycosylation level might be the theoretic mechanism of AMI in the treatment of IgAN patients.

Currently, IgA nephropathy was mainly treated with RAS inhibitors, corticosteroids and immunosuppressant, which is not specific and sometimes ineffective [15]. Immunosuppressant treatment sometimes even cause severe side effect such as life-threating infection. Moreover, the anti-hypertensive effect of RAS inhibitors limited its uses in patients with normal blood pressure. Astagalus membranaceus, as a widely used immunomodulating herb in traditional Chinese medicine, has been recognized as a helpful complementary therapy of IgAN. Our current study provided new evidence of AM in the treatment of IgAN patients. However, further random control trials should be carried out to verify the in vitro results in IgAN patients.

## Conclusion

In summary, for the first time, our data demonstrates that Astagalus membranaceus injection could up-regulate cosmc gene expression and improve IgA1 O-glycosylation level of peripheral B lymphocyte from IgAN in vitro, which may be the potential mechanism of its therapeutic effect in IgAN.

## Competing interests

The authors declare that they have no competing interests.

## Authors’ contributions

LJ carried out the study, recruited patients, wrote manuscript. XLC performed Western Blot and ELISA assay. XZ performed realtime PCR assay. ZL recruited patients. LCY recruited patients. JMF participated in study design. WXT performed data analysis. WQ designed, analyzed and interpreted data and revised manuscript. All authors read and approved the final manuscript.

## Authors’ information

LJ, XLC, ZL, LCY, WXT and WQ are faculty/staff of West China Hospital of Sichuan University. XZ is staff of Sichuan Provincial People’s Hospital. JMF is professor of State Key Laboratory of Biotherapy of Sichuan University. ZL, LCY and WQ are Assistant Professors, WXT and JMF are Professors.

## Pre-publication history

The pre-publication history for this paper can be accessed here:

http://www.biomedcentral.com/1472-6882/14/195/prepub
